# Supplementary material for: Work-related COVID-19 transmission in six Asian countries/areas: A follow-up study
Source: PLoS One. 2020 May 19;15(5):e0233588. doi: 10.1371/journal.pone.0233588 (PMC7237000; doi:10.1371/journal.pone.0233588)
Supplement: S1 Table — (DOCX) [file pone.0233588.s001.docx]

**S1 Table. Information of the 103 possible work-related COVID-19 cases during the study period.**

| **Country / Area** | **Occupation (ISCO-08)** | **Released Date** | **Type** |
| --- | --- | --- | --- |
| Taiwan | Car, taxi and van drivers | 20200216 | Unknown transmission source; no obvious contact history but likely to be infected in the working environment |
| Taiwan | Personal care workers in health services | 20200226 | Close contact with a confirmed case due to work |
| Taiwan | Domestic cleaners and helpers | 20200229 | Close contact with a confirmed case due to work |
| Taiwan | Nursing professionals | 20200229 | Close contact with a confirmed case due to work |
| Taiwan | Nursing professionals | 20200229 | Close contact with a confirmed case due to work |
| Taiwan | Nursing professionals | 20200229 | Close contact with a confirmed case due to work |
| Hongkong | Shop salespersons | 20200204 | Unknown transmission source; no obvious contact history but likely to be infected in the working environment |
| Hongkong | Religious professionals | 20200211 | Unknown transmission source; no obvious contact history but likely to be infected in the working environment |
| Hongkong | Domestic cleaners and helpers | 20200211 | Close contact with a confirmed case due to work |
| Hongkong | Cooks | 20200212 | Close contact with a confirmed case due to work |
| Hongkong | Waiter or bartenders | 20200214 | Unknown transmission source; no obvious contact history but likely to be infected in the working environment |
| Hongkong | unspecified | 20200217 | Close contact with a confirmed case due to work |
| Hongkong | Domestic housekeepers | 20200218 | Close contact with a confirmed case due to work |
| Hongkong | Car, taxi and van drivers | 20200220 | Unknown transmission source; no obvious contact history but likely to be infected in the working environment |
| Hongkong | Police officers | 20200220 | Unknown transmission source; no obvious contact history but likely to be infected in the working environment |
| Hongkong | Security guards | 20200223 | Unknown transmission source; no obvious contact history but likely to be infected in the working environment |
| Hongkong | Religious professionals | 20200304 | Unknown transmission source; no obvious contact history but likely to be infected in the working environment |
| Hongkong | Domestic housekeepers | 20200304 | Close contact with a confirmed case due to work |
| Hongkong | Domestic housekeepers | 20200308 | Close contact with a confirmed case due to work |
| Hongkong | Car, taxi and van drivers | 20200309 | Close contact with a confirmed case due to work |
| Thailand | Car, taxi and van drivers | 20200131 | Unknown transmission source; no obvious contact history but likely to be infected in the working environment |
| Thailand | Car, taxi and van drivers | 20200204 | Unknown transmission source; no obvious contact history but likely to be infected in the working environment |
| Thailand | Car, taxi and van drivers | 20200204 | Unknown transmission source; no obvious contact history but likely to be infected in the working environment |
| Thailand | Health professionals | 20200214 | Close contact with a confirmed case due to work |
| Thailand | Travel attendants, conductors and guides | 20200225 | Unknown transmission source; no obvious contact history but likely to be infected in the working environment |
| Thailand | Shop salespersons | 20200229 | Unknown transmission source; no obvious contact history but likely to be infected in the working environment |
| Thailand | Travel attendants, conductors and guides | 20200302 | Close contact with a confirmed case due to work |
| Vietnam | Receptionists | 20200201 | Close contact with a confirmed case due to work |
| Japan | Bus and tram drivers | 20200128 | Close contact with a confirmed case due to work. |
| Japan | Travel attendants, conductors and guides | 20200129 | Close contact with a confirmed case due to work. |
| Japan | Bus and tram drivers | 20200131 | Close contact with a confirmed case due to work. |
| Japan | Travel attendants, conductors and guides | 20200205 | Unknown transmission source: no obvious contact history but likely to be infected in the working environment. |
| Japan | Car, taxi and van drivers | 20200213 | Unknown transmission source: no obvious contact history but likely to be infected in the working environment. |
| Japan | Medical doctors | 20200213 | Unknown transmission source: no obvious contact history but likely to be infected in the working environment. |
| Japan | Car, taxi and van drivers | 20200214 | Close contact with a confirmed case due to work. |
| Japan | Car, taxi and van drivers | 20200214 | Close contact with a confirmed case due to work. |
| Japan | Health professionals | 20200215 | Unknown transmission source: no obvious contact history but likely to be infected in the working environment. |
| Japan | Nursing professionals | 20200217 | Close contact with a confirmed case due to work. |
| Japan | Health professionals | 20200218 | Close contact with a confirmed case due to work. |
| Japan | Car, taxi and van drivers | 20200219 | Unknown transmission source: no obvious contact history but likely to be infected in the working environment. |
| Japan | Car, taxi and van drivers | 20200219 | Close contact with a confirmed case due to work. |
| Japan | Police officer | 20200221 | Unknown transmission source: no obvious contact history but likely to be infected in the working environment. |
| Japan | Nursing professionals | 20200222 | Unknown transmission source: no obvious contact history but likely to be infected in the working environment. |
| Japan | Nursing professionals | 20200222 | Close contact with a confirmed case due to work. |
| Japan | Locomotive engine drivers and related workers | 20200222 | Unknown transmission source: no obvious contact history but likely to be infected in the working environment. |
| Japan | Fire fighter | 20200223 | Unknown transmission source: no obvious contact history but likely to be infected in the working environment. |
| Japan | Medical doctors | 20200226 | Close contact with a confirmed case due to work. |
| Japan | Medical doctors | 20200226 | Unknown transmission source: no obvious contact history but likely to be infected in the working environment. |
| Japan | Locomotive engine drivers and related workers | 20200226 | Unknown transmission source: no obvious contact history but likely to be infected in the working environment. |
| Japan | Fire fighter | 20200227 | Unknown transmission source: no obvious contact history but likely to be infected in the working environment. |
| Japan | Nursing professionals | 20200227 | Close contact with a confirmed case due to work. |
| Japan | Health professionals | 20200229 | Unknown transmission source: no obvious contact history but likely to be infected in the working environment. |
| Japan | Health professionals | 20200229 | Unknown transmission source: no obvious contact history but likely to be infected in the working environment. |
| Japan | Car, taxi and van drivers | 20200301 | Unknown transmission source: no obvious contact history but likely to be infected in the working environment. |
| Japan | Nursing professionals | 20200301 | Unknown transmission source: no obvious contact history but likely to be infected in the working environment. |
| Japan | Health professionals | 20200301 | Unknown transmission source: no obvious contact history but likely to be infected in the working environment. |
| Japan | Health professionals | 20200302 | Unknown transmission source: no obvious contact history but likely to be infected in the working environment. |
| Japan | Nursing professionals | 20200302 | Unknown transmission source: no obvious contact history but likely to be infected in the working environment. |
| Japan | Waiter or bartenders | 20200303 | Unknown transmission source: no obvious contact history but likely to be infected in the working environment. |
| Japan | Nursing professionals | 20200304 | Close contact with a confirmed case due to work. |
| Japan | Health professionals | 20200304 | Close contact with a confirmed case due to work. |
| Japan | Police officer | 20200305 | Unknown transmission source: no obvious contact history but likely to be infected in the working environment. |
| Japan | Health professionals | 20200306 | Close contact with a confirmed case due to work. |
| Singapore | Shop salespersons | 20200201 | Unknown transmission source; no obvious contact history but likely to be infected in the working environment |
| Singapore | Shop salespersons | 20200204 | Unknown transmission source; no obvious contact history but likely to be infected in the working environment |
| Singapore | Domestic housekeepers | 20200204 | Close contact with a confirmed case due to work |
| Singapore | Travel attendants, conductors and guides | 20200204 | Unknown transmission source; no obvious contact history but likely to be infected in the working environment |
| Singapore | Shop salespersons | 20200205 | Unknown transmission source; no obvious contact history but likely to be infected in the working environment |
| Singapore | unspecified | 20200206 | Unknown transmission source; no obvious contact history but likely to be infected in the working environment |
| Singapore | Shop salespersons | 20200208 | Unknown transmission source; no obvious contact history but likely to be infected in the working environment |
| Singapore | Car, taxi and van drivers | 20200208 | Unknown transmission source; no obvious contact history but likely to be infected in the working environment |
| Singapore | unspecified | 20200208 | Unknown transmission source; no obvious contact history but likely to be infected in the working environment |
| Singapore | Car, taxi and van drivers | 20200208 | Unknown transmission source; no obvious contact history but likely to be infected in the working environment |
| Singapore | unspecified | 20200208 | Unknown transmission source; no obvious contact history but likely to be infected in the working environment |
| Singapore | Shop salespersons | 20200208 | Unknown transmission source; no obvious contact history but likely to be infected in the working environment |
| Singapore | Construction labourers | 20200209 | Unknown transmission source; no obvious contact history but likely to be infected in the working environment |
| Singapore | Receptionists | 20200209 | Unknown transmission source; no obvious contact history but likely to be infected in the working environment |
| Singapore | Security guards | 20200210 | Unknown transmission source; no obvious contact history but likely to be infected in the working environment |
| Singapore | Receptionists | 20200211 | Unknown transmission source; no obvious contact history but likely to be infected in the working environment |
| Singapore | Construction labourers | 20200211 | Unknown transmission source; no obvious contact history but likely to be infected in the working environment |
| Singapore | Religious professionals | 20200212 | Unknown transmission source; no obvious contact history but likely to be infected in the working environment |
| Singapore | Religious professionals | 20200212 | Unknown transmission source; no obvious contact history but likely to be infected in the working environment |
| Singapore | Religious professionals | 20200213 | Unknown transmission source; no obvious contact history but likely to be infected in the working environment |
| Singapore | Construction labourers | 20200213 | Unknown transmission source; no obvious contact history but likely to be infected in the working environment |
| Singapore | Construction labourers | 20200213 | Unknown transmission source; no obvious contact history but likely to be infected in the working environment |
| Singapore | Health professionals | 20200214 | Unknown transmission source; no obvious contact history but likely to be infected in the working environment |
| Singapore | Car, taxi and van drivers | 20200214 | Unknown transmission source; no obvious contact history but likely to be infected in the working environment |
| Singapore | Religious professionals | 20200214 | Unknown transmission source; no obvious contact history but likely to be infected in the working environment |
| Singapore | Construction labourers | 20200215 | Unknown transmission source; no obvious contact history but likely to be infected in the working environment |
| Singapore | unspecified | 20200226 | Unknown transmission source; no obvious contact history but likely to be infected in the working environment |
| Singapore | unspecified | 20200227 | Unknown transmission source; no obvious contact history but likely to be infected in the working environment |
| Singapore | unspecified | 20200228 | Unknown transmission source; no obvious contact history but likely to be infected in the working environment |
| Singapore | unspecified | 20200228 | Unknown transmission source; no obvious contact history but likely to be infected in the working environment |
| Singapore | unspecified | 20200229 | Close contact with a confirmed case due to work |
| Singapore | unspecified | 20200229 | Close contact with a confirmed case due to work |
| Singapore | unspecified | 20200229 | Close contact with a confirmed case due to work |
| Singapore | Domestic housekeepers | 20200229 | Close contact with a confirmed case due to work |
| Singapore | unspecified | 20200301 | Unknown transmission source; no obvious contact history but likely to be infected in the working environment |
| Singapore | Domestic housekeepers | 20200301 | Close contact with a confirmed case due to work |
| Singapore | unspecified | 20200301 | Unknown transmission source; no obvious contact history but likely to be infected in the working environment |
| Singapore | unspecified | 20200301 | Unknown transmission source; no obvious contact history but likely to be infected in the working environment |
| Singapore | Domestic housekeepers | 20200302 | Close contact with a confirmed case due to work |
| Singapore | unspecified | 20200303 | Unknown transmission source; no obvious contact history but likely to be infected in the working environment |

ISCO-08: International Standard Classification of Occupations, 2008
